# Reporting quality of randomized controlled trials in the *Journal of Korean Biological Nursing Science* (2011~2024): an evaluation based on the CONSORT 2025 guidelines

**DOI:** 10.7586/jkbns.25.063

**Published:** 2025-11-26

**Authors:** Mi-Kyoung Cho, Mi Young Kim

**Affiliations:** 1 Department of Nursing Science, College of Nursing, Chungbuk National University, Cheongju, Korea; 2 College of Nursing, Hanyang University, Seoul, Korea

**Keywords:** Randomized controlled trial, Guideline adherence, Quality control, Nursing research

## Abstract

**Purpose:**

This study aims to evaluate the reporting completeness of randomized controlled trials (RCTs) published in the *Journal of Korean Biological Nursing Science* (JKBNS) from 2011 to 2024 using the updated Consolidated Standards of Reporting Trials (CONSORT) 2025 guidelines.

**Methods:**

A systematic search of the JKBNS archive was conducted using keywords related to random allocation. A total of 22 RCTs were identified and included in this methodological review. Eligible studies were defined as RCTs involving human participants. Reporting completeness was assessed using the CONSORT 2025 checklist. In addition, a conceptual mapping analysis was performed to examine the alignment between CONSORT 2025 checklist items and methodological quality domains derived from the Risk of Bias 2.0 (RoB 2.0), the Scottish Intercollegiate Guidelines Network (SIGN), and the Joanna Briggs Institute (JBI) critical appraisal tools.

**Results:**

Of the 22 reviewed RCTs, 19 used a parallel-group design and 3 employed a crossover design. Most studies evaluated non-pharmacological interventions, most commonly aromatherapy. The mean CONSORT 2025 compliance score was 24.77 ± 3.15, with 18 items consistently reported across studies. However, critical elements such as protocol amendments, interim analyses, and approaches to handling missing data were rarely addressed. Evaluation of 42 detailed items corresponding to 30 checklist numbers showed an overall average reporting rate of 59.0%. Fourteen CONSORT 2025 checklist items demonstrated conceptual alignment with domains from RoB 2.0, SIGN, and JBI.

**Conclusion:**

The overall reporting quality of RCTs published in JKBNS was moderate. Future RCT submissions to nursing journals should emphasize rigorous and comprehensive adherence to reporting standards such as the CONSORT 2025 checklist to improve methodological transparency and reproducibility.

## INTRODUCTION

### 1. Background

Nursing is an applied science based on the humanities, natural sciences, and social sciences, with the goal of education being the cultivation of professional nurses. Nursing focuses on promoting health and improving individuals' quality of life. It is grounded in scientific evidence to fulfill its professional responsibilities in an ever-changing healthcare environment. To realize professional nursing practice and evidence based practice, basic nursing science has been emphasized as a foundational discipline of nursing, with its educational value and necessity continuously increasing.

As the importance of this foundational discipline has become increasingly recognized, the Korean Society of Biological Nursing Science published the first issue of the *Journal of Korean Biological Nursing Science* (JKBNS) in December 1999 to establish the field's academic standing. Since then, the journal has developed a systematic structure as a scholarly publication, promoted academic exchange, and built a knowledge base. In 2010, the journal was indexed in the Korea Citation Index, and in 2012, it was listed in the Cumulative Index to Nursing and Allied Health Literature (CINAHL). It has since maintained its academic credibility through continuous indexing in CINAHL for 12 years. Furthermore, by meeting strict evaluation criteria, the journal has been indexed in Scopus, an internationally recognized database, thus elevating its status to that of a globally acknowledged academic journal [1]. This milestone has enabled researchers worldwide to access the journal’s articles, marking a significant turning point in its growth into a world class scholarly journal in the field of basic nursing science.

As part of efforts to promote the growth of an academic journal, analyzing the articles published in the journal can help identify specific strategies to improve its quality. In this regard, a previous study examined 179 articles published in the JKBNS from its inaugural issue in 1999 through 2010 [2]. The study reported an increase in the number of articles published annually, diversification of research topics and methodologies, and a higher proportion of experimental studies compared to other nursing journals.

Since then, numerous articles have been published. Moving forward, for the journal to achieve qualitative growth, it is essential not only to meet the evaluation criteria set by the Web of Science Group but also to include studies that demonstrate originality, academic and practical applicability, and both regional and international significance [3].

In recent years, in the field of nursing, there has been increasing emphasis on adherence to research reporting guidelines to improve research quality and enhance the reliability of research outcomes. Since the 1990s, various research groups have developed standardized reporting guidelines for different study designs by identifying essential items that should be included in research reports to improve reporting quality [4]. These guidelines have been promoted and implemented by international journals and research institutions.

However, to date, there has been a lack of systematic analysis regarding the extent to which articles published in the JKBNS comply with international reporting guidelines appropriate for their respective study designs. Therefore, it is now necessary to evaluate article quality against reporting guidelines and propose practical strategies for improvement, thereby enhancing article quality in the journal.

For the qualitative development of an academic journal, it is essential not only to increase the number of published articles but also to review study designs and levels of evidence systematically. Among various study designs, randomized controlled trials (RCTs) are considered among the highest levels of evidence and play a crucial role in supporting reliable clinical and academic decision-making. Consequently, when analyzing a journal's research trends, prioritizing the review of RCTs is essential to advancing the journal's qualitative quality.

Therefore, this study aims to conduct a systematic analysis of RCTs published in the JKBNS to derive specific improvement strategies and research design plans to enhance the journal's quality. By prioritizing the review of studies with a high level of evidence, it will be possible not only to meet journal evaluation criteria but also to suggest research directions that can make practical contributions to future clinical and academic practice.

Accordingly, this study targets RCTs published in the JKBNS between 2011 and 2024. It seeks to objectively evaluate their quality by applying the Consolidated Standards of Reporting Trials (CONSORT), an international reporting guideline for RCTs. The CONSORT guideline, which serves as a representative reporting standard for RCTs, was first introduced in 1996 and subsequently revised in 2001 and 2010 [5]. Most recently, the 2025 version was released, adding new items and revising or integrating existing ones to enhance transparency and reproducibility in research reporting [6].

In particular, the 2025 version introduced seven newly added items: data sharing, patient and public involvement, detailed descriptions of how the intervention and comparator were actually implemented, reporting of adverse events and unintended effects, characteristics of participants and study sites, as well as information on the number of participants and availability of data at each time point. Additionally, several existing items were strengthened, including reporting the accessibility of the study protocol and statistical analysis plan, documentation of any changes to outcomes or analyses after trial initiation, and reporting the number of participants included for each outcome variable. Meanwhile, the previously separate item on generalisability was removed and integrated into the limitations section. Furthermore, the revised version reflects the movement toward open science, emphasizing openness and accountability in research through requirements such as registration, data and code sharing, and conflict of interest reporting. With these updates, CONSORT 2025 presents a more rigorous and comprehensive international standard for clinical trial reporting [6].

In addition, systematically evaluating research quality and risk of bias in clinical research and evidence based practice has recently become a crucial process for interpreting study results and determining their applicability. In this study, three tools were used to assess RCTs and various study designs: Cochrane’s Risk of Bias 2.0 (RoB 2.0), the Scottish Intercollegiate Guidelines Network (SIGN), and the Joanna Briggs Institute (JBI) critical appraisal tools. RoB 2, developed by Cochrane, is a risk of bias assessment tool specifically for RCTs. It evaluates five domains — randomization process, intervention implementation, outcome measurement, and selective reporting — to determine the reliability of study results [7]. The SIGN checklist can be applied not only to RCTs but also to other study designs, such as cohort studies and case-control studies, grading the quality of research based on the appropriateness of study design and analysis [8]. The JBI tools provide customized checklists for nearly all study designs, including RCTs, cohort studies, and qualitative research, allowing for a comprehensive evaluation of the quality of evidence [9].

The CONSORT 2025 guidelines focus primarily on the completeness of reporting and do not directly assess methodological reliability, such as the validity of the study design, control of bias, or appropriateness of data analysis [10]. While the quality of reporting reflects how clearly and systematically researchers describe the study procedures and results, methodological reliability (or methodological quality) evaluates the internal validity of the study and the extent to which systematic biases are controlled. In other words, strict adherence to CONSORT does not necessarily guarantee high methodological quality; instead, these two assessments are conceptually distinct yet complementary. In this study, we aim not only to evaluate adherence to the CONSORT 2025 reporting guidelines but also to map these items against established quality assessment tools to provide a more comprehensive assessment of study rigor. This study aimed to objectively evaluate the quality of articles by applying the CONSORT, an international reporting guideline appropriate for RCTs, to RCTs published in the JKBNS between 2011 and 2024. Through this process, the study seeks to provide foundational data to strengthen the journal’s quality management system and to explore future directions for the qualitative development of the journal.

### 2. Study aim

The purpose of this study is to systematically evaluate the quality of RCTs published in the JKBNS according to international standards. The specific objectives are as follows:

1) To examine the characteristics of RCTs published in JKBNS between 2011 and 2024.

2) To assess the reporting quality of these RCTs based on the CONSORT 2025 checklist.

3) To map the CONSORT 2025 checklist against established international appraisal tools—RoB 2.0, SIGN, and JBI—in order to identify areas of convergence and inform future quality enhancement efforts.

## METHODS

### 1. Study design

This study is a methodological study designed to evaluate the quality of academic articles systematically.

### 2. Samples

**Figure 1.**
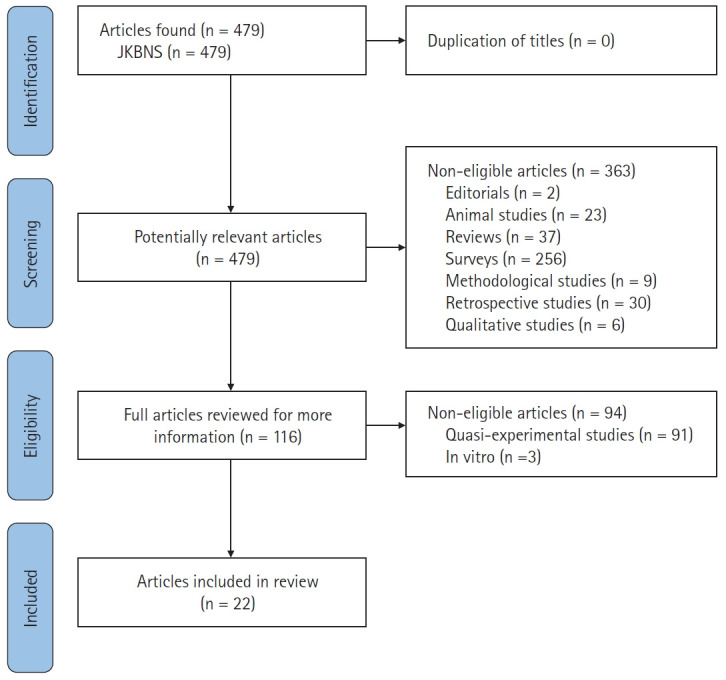
Flow chart of study selection. JKBNS = *Journal of Korean Biological Nursing Science*.

The data for this study consisted of RCTs published in JKBNS between January 2011 and December 2024. The inclusion criteria were original research articles involving human participants that explicitly identified the study as an RCT in the title, abstract, or methods section. The exclusion criteria included editorials, animal studies, reviews or meta-analyses, surveys, methodological studies, qualitative studies, retrospective studies, and quasi-experimental studies. Based on these criteria, 22 studies were finally included (Figure 1).

### 3. Instruments

The CONSORT 2025 checklist was used to evaluate whether the reporting of the included RCTs adhered to established reporting standards. In addition, three validated quality appraisal tools—RoB 2.0, SIGN checklist for RCTs, and JBI critical appraisal checklist—were used to map their respective items to the corresponding components of the CONSORT 2025 checklist.

#### 1) The CONSORT 2025 checklist

The CONSORT 2025 checklist is the latest revised reporting guideline aimed at ensuring clarity, transparency, and completeness in the reporting process of RCTs [6]. This checklist comprises six structured sections that cover the entire research article, including title and abstract, introduction, methods, results, discussion, and open science. Each section includes detailed items that specify the minimum requirements for proper reporting. Key evaluation items include whether the study type is clearly stated in the title and abstract section, whether a structured abstract is provided, whether the background and objectives are clearly described in the introduction section, and whether the methods section includes methodological components such as trial design, eligibility criteria, intervention details, outcome measures, sample size calculation, randomization procedures, allocation concealment, and blinding strategies. The results section includes items related to participant flow, recruitment and follow-up periods, participant characteristics, primary and secondary outcomes, and appropriateness of statistical analysis. The discussion section focuses on the interpretation of findings, study limitations, and the external validity of the results. In contrast, the open science section includes items such as trial registration, funding sources, ethical approval, accessibility of the study protocol, and data sharing plans. This checklist consists of 30 items. Compared to the 2010 version, the 2025 revision includes newly added items and modifications that reflect contemporary expectations in clinical research, such as stakeholder involvement, equity considerations, and transparency in data processing and sharing. In this study, each included RCTs article was evaluated for compliance with each item of the CONSORT 2025 checklist. All items were scored using a binary coding system. If the item was explicitly described in the article, it was scored as “1”; if it was not described or was unclear, it was scored as “0”. If an item was entirely unaddressed, it was classified as “not reported” and scored as 0. In cases where certain checklist items were not applicable due to study design characteristics (e.g., crossover trials or single-group studies), those items were also scored as 0.

#### 2) The Cochrane RoB 2.0

The RoB 2.0 tool is a rigorously developed, widely used instrument for evaluating the internal validity of RCTs. Developed by the Cochrane Collaboration, this tool assesses the risk of bias across five domains related to the design, conduct, and reporting of clinical trials [7]. The five domains are randomization process, deviations from intended interventions, missing outcome data, measurement of outcomes, and selection of the reported results. Specifically, it evaluates the adequacy of random sequence generation and allocation concealment, the blinding of participants and investigators, adherence to intervention protocols, handling of missing data, appropriateness and blinding of outcome measurement methods, and the presence of selective outcome reporting. The overall risk of bias is determined not by a scoring system but by a qualitative judgment that synthesizes the assessments across the five domains. For this purpose, each domain provides structured signaling questions, and reviewers respond with “Yes”, “Probably Yes”, “Probably No”, “No”, or “No Information”. Based on these responses, the risk of bias for each domain is assessed, and each study is ultimately classified as having an overall risk of bias of “low”, “some concerns”, or “high”.

#### 3) The SIGN checklist for RCTs

The SIGN checklist for RCTs is a methodological tool developed to systematically assess the internal validity and methodological rigor of clinical research. This checklist is designed to identify potential sources of bias across the design, conduct, and analysis phases of RCTs and to determine the level and reliability of clinical evidence [8]. Key evaluation criteria include the appropriateness of random sequence generation and allocation concealment, baseline comparability between groups, clarity in the description of interventions and control conditions, and blinding of participants and researchers. In addition, it assesses the completeness of follow-up and handling of missing data, the validity and consistency of outcome measurements, adherence to prespecified analysis plans (e.g., analysis of prespecified outcomes), the use of intention-to-treat (ITT) analysis, and the justification and adequacy of sample size and statistical methods. Based on the degree to which these criteria are satisfied, each study is classified as providing high-quality evidence (++), acceptable-quality evidence (+), or low-quality evidence (0).

#### 4) The JBI critical appraisal checklist

The JBI critical appraisal checklist for RCTs is a tool designed to evaluate the appropriateness of the study design and the methodological consistency of trial implementation. This checklist is designed to assess whether randomized clinical trials provide reliable evidence regarding internal validity and feasibility and is widely used as a key instrument in evidence-based nursing and healthcare practice [9]. The JBI checklist consists of 13 items, which cover key elements such as whether randomization was performed correctly, whether allocation was adequately concealed, the baseline comparability between intervention and control groups, clarity of intervention descriptions, blinding of participants, researchers, and outcome assessors, completeness of follow-up, application of ITT analysis, reliability and validity of outcome measures, and appropriateness of statistical analysis. Each item is assessed using one of four response options: “Yes”, “No”, “Unclear”, or “Not applicable”.

### 4. Data collection

Data were retrieved from the archive of the JKBNS website using the keywords “randomized,” “무작위,” and “RCTs.” The search was conducted between June 13 and June 25, 2025. The identified articles were organized in an Excel spreadsheet containing the author(s), year of publication, article title, and DOI. From July 13 to August 9, 2025, two independent reviewers (Cho and Kim) screened the titles, study designs, and full texts of the retrieved articles. In the first stage, studies were selected based on titles containing terms such as “실험연구” (experimental study), “무작위” (randomized), “random*,” “RCTs,” and “효과” (effect). In the second stage, abstracts were reviewed to identify studies explicitly mentioning “RCTs” or “randomization.” In the third stage, articles labeled as “experimental study” or “quasi-experimental study” in the methods section were further assessed. If random allocation was clearly described in the full text, they were classified as RCTs and included in the final sample. Disagreements regarding study inclusion primarily concerned whether to include crossover RCTs or post-test-only RCTs. After discussion, both types of studies were agreed to be included.

### 5. Data analysis

Data analysis was conducted to systematically summarize the general characteristics of the included RCTs and report their quality, as well as to compare the conceptual concordance and differences among the assessment tools. The general characteristics of the studies (authors, year of publication, study design, method of randomization, participant characteristics, sample size, type of intervention, and outcome variables) were summarized from the original articles. Reporting quality was assessed according to the 30 items of the CONSORT 2025 checklist. Each item was coded as "1" if explicitly reported or "0" if not reported or unclear. Compliance with individual items was calculated as frequencies, and the total and mean scores for each study were computed to evaluate the overall reporting quality. Two independent reviewers, both experienced in conducting meta-analyses and quality appraisals, performed the evaluations. Before the formal assessment, they held preliminary discussions to align their understanding of the evaluation criteria and conducted a pilot assessment on two to three studies to calibrate scoring. As a result, inter-rater reliability, assessed using Cohen’s kappa, indicated perfect agreement (κ = 1.00; 95% CI: 1.00 to 1.00) across all assessments. In addition, conceptual mapping was performed between the CONSORT 2025 items and the domains of the Cochrane RoB 2.0, the SIGN checklist for RCTs, and the JBI critical appraisal checklist to identify the correspondence across the tools. This mapping was carried out based on two rounds of expert consensus. The researchers with expertise in evidence based practice and guideline adaptation independently reviewed the definitions and scope of each item across tools. Subsequently, consensus meetings were held to reconcile interpretations and resolve discrepancies. Only items with apparent conceptual equivalence were retained in the final mapping framework.

### 6. Ethical considerations

This study is a literature analysis focusing on previously published journal articles. During the analysis, researchers made every effort to minimize subjective bias, and all data were obtained from peer-reviewed publications, with sources clearly cited. Academic and publication ethics were strictly followed throughout the study process and reporting of results.

## RESULTS

### 1. Characteristics of RCTs in JKBNS (2011~2024)

This study analyzed the characteristics of 22 RCTs published in the JKBNS between 2011 and 2024. Of these, 19 were standard RCTs, and 3 employed a crossover RCT design. Although approximately three RCTs were published annually, no RCT articles were published in 2016, 2020, 2022, or 2023, and only one RCT was published in each of 2017, 2018, and 2021. Randomization methods were reported in 19 studies, using diverse techniques such as Excel based randomization, computer generated random numbers, random number tables, coin tossing, and lottery methods. However, three studies did not specify their randomization procedures. Participants included university students, nurses, surgical patients, individuals with chronic diseases, and older adults, with sample sizes ranging from 18 to 132. Most interventions were non-pharmacological, with aromatherapy being the most frequent (eight studies), followed by music therapy (three studies) and hand hygiene interventions (two studies). Intervention durations ranged from a single day to 12 weeks, with one study not reporting the intervention duration. The number of intervention sessions varied from 1 to 42, and 14 studies implemented a single-session intervention. The time per session ranged from 15 seconds to two weeks, though some studies reported session time in relative terms such as "until the end of surgery," "until recovery room discharge," or "continuous"; five studies did not report session time. In eight studies, the control group received no intervention, while three used usual or traditional care. Other comparators included almond oil inhalation, natural hand drying, saline, and rest. Different outcome variables were assessed, covering a range of domains, including pain, physiological parameters (e.g., blood pressure, pulse, respiration, heart rate variability), psychological indicators (e.g., anxiety, stress, sleep disturbance, depression), and bacterial counts, thereby capturing the multidimensional effects of nursing interventions (Table 1).

A total of 22 RCTs published in JKBNS between 2011 and 2024 were analyzed to assess reporting completeness according to the CONSORT guidelines. Evaluation of 42 detailed items corresponding to 30 checklist numbers revealed an overall average reporting rate of 59.0%.

### 2. Reporting of RCTs based on the CONSORT 2025 in JKBNS (2011~2024)

Based on an item-by-item analysis of CONSORT 2025, the following components were reported in all included studies: '1b. Structured summary of the trial design, methods, results, and conclusions', '6. Scientific background and rationale', '7. Specific objectives, '9. Trial design', '11. Trial setting', '12a. Eligibility criteria for participants', '12b. Eligibility criteria for sites/providers', '13. Intervention and comparator details', '14. Prespecified outcomes', '19. Implementation', '21b. Definition of analysis population', '22b. Losses and exclusions', '23a. Recruitment and follow-up dates', '24a. Intervention delivery', '26. Outcomes and estimation', '29. Interpretation', and '30. Limitations'. Item '16a. Sample size determination' was adequately reported in 21 studies, while items '17a. Sequence generation' and '21a. Statistical methods' were sufficiently addressed in 20 studies. In contrast, items such as '10. Changes to trial protocol', '16b. Interim analyses and stopping guidelines', '21c. Missing data handling', and '23b. Reason for trial ending' were not reported in any of the reviewed studies. Additionally, items '21d. Additional analyses' (Study ID: 9), '27. Harms' (Study ID: 18), and '28. Ancillary analyses' (Study ID: 1) were each reported in only one study, respectively (Table 2).

Since 2005, the International Committee of Medical Journal Editors (ICMJE) [11], and since 2013, the Declaration of Helsinki [12], as well as Dickersin & Rennie [13], have emphasized the importance of clinical trial registration. Dickersin and Rennie stated that researchers, research institutions and organizations, journal editors, legislators, and consumers — in fact, all stakeholders — must take immediate action, both collectively and within their respective domains, to ensure comprehensive registration of clinical trials. The Korean Association of Medical Journal Editors [14] reflected the principles of the ICMJE in its Korean-translated revised editions, distributed in February 2006 and September 2008, of the Uniform Requirements for Manuscripts Submitted to Biomedical Journals: Writing and Editing for Biomedical Publication. These guidelines required that the clinical trial registration number be included at the end of the abstract. In 2010, the Clinical Research Information Service (CRIS) was launched as a system for registering clinical trials. By May of that year, CRIS officially joined the World Health Organization International Clinical Trials Registry Platform as a primary registry, establishing a registration system that met international standards [15]. In 2024 JKBNS specified in the '8. Ethical considerations' section of RCT articles that RCT protocol registration must be reported [16].

### 3. Trend analysis across combined years of studies published in JKBNS (2011~2024) based on CONSORT 2025 guidelines

The changes observed over five-year intervals are summarized in Table 3. A closer examination revealed that certain items consistently demonstrated high ('1b. Structured summary of the trial design, methods, results, and conclusions','6. Scientific background and rationale', '7. Specific objectives', and related items) or low ('10. Changes to trial protocol','16b. Interim analyses and stopping guidelines', and similar items) reporting rates across all periods.

Some items showed a progressive increase in reporting rates in more recent years ('1a. Identification as a randomized trial in the title', '2. Trial registration', '3. Protocol and statistical analysis plan', '4. Data sharing', '5b. Conflicts of interest', '18. Allocation concealment mechanism', and related items). Conversely, a few items exhibited a declining trend in reporting over time ('24 b. Concomitant care', '25. Baseline data', and similar items).

### 4. Mapping the CONSORT 2025 checklist to RoB 2.0, SIGN, and JBI: a comparative analysis of RCT quality criteria

The CONSORT 2025 checklist was mapped against the quality assessment tools for RCTs, including the Cochrane RoB 2.0, the SIGN, and the JBI instruments (Table 4). A total of 14 items from the CONSORT 2025 checklist were found to be commonly matched with these three appraisal tools, reflecting core elements of RCT design. These items are directly related to ensuring the internal validity of trials and include: prespecified outcomes (Item 14), random sequence generation (Items 17a and 17b), allocation concealment (Item 18), blinding (Items 20a and 20b), definition of the analysis population (Item 21b), handling of missing data (Item 21c), participant flow (Item 22a), losses and exclusions (Item 22b), intervention delivery (Item 24a), concomitant care (Item 24b), baseline data (Item 25), and outcomes and estimation (Item 26).

Specifically, Item 14 “Prespecified outcomes” was matched with RoB 2.0 items 4.1, 4.2, 5.2, and 5.3, which assess the standardization and reliability of outcome measurements and the consistency of analysis. It was also matched with SIGN item 1.7 and JBI items 8 and 9. Items 17a “Sequence generation” and 17b “Type of randomization and restriction” were mapped to RoB 2.0 item 1.1, SIGN items 1.2 and 2.1, and JBI item 1, which evaluate the adequacy of the randomization procedure. Item 18 “Allocation concealment mechanism” was matched with RoB 2.0 item 1.2, SIGN items 1.3 and 2.1, and JBI item 2, focusing on whether the allocation was adequately concealed prior to assignment. Items 20a “Who was blinded” and 20b “How blinding was achieved” were matched with RoB 2.0 items 2.1, 2.2, and 4.3; SIGN items 1.4 and 2.1; and JBI items 4, 5, and 7, all assessing blinding among participants, providers, and outcome assessors. Item 21b “Definition of analysis population” was matched with RoB 2.0 item 2.6, SIGN item 1.9, and JBI item 11, evaluating whether the analyzed population was defined in accordance with the pre-specified analysis plan. Item 21c “Missing data handling” was matched with RoB 2.0 items 3.1~3.4, SIGN item 2.1, and JBI item 10, focusing on the appropriateness of handling missing data and potential bias. Item 22a “Participant flow” was matched with RoB 2.0 item 3.1, SIGN item 2.1, and JBI item 10, assessing transparency in reporting participant movement, including recruitment and analysis inclusion. Item 22b “Losses and exclusions” was matched with RoB 2.0 items 3.1~3.4, SIGN items 1.8 and 2.1, and JBI item 10, focusing on the reporting and justification of participant withdrawals or exclusions. Item 24a “Intervention delivery” was matched with RoB 2.0 items 2.3~2.5, SIGN item 1.6, and JBI item 6, examining whether the intervention was delivered as planned. Item 24b “Concomitant care” was similarly matched with RoB 2.0 items 2.3~2.5, SIGN item 1.6, and JBI item 6, assessing the balance of concomitant treatments between groups. Item 25 “Baseline data” was matched with RoB 2.0 item 1.3, SIGN items 1.5 and 2.1, and JBI item 3, evaluating comparability of baseline characteristics. Finally, Item 26 “Outcomes and estimation” was matched with RoB 2.0 items 4.1~4.5, SIGN item 2.1, and JBI items 8 and 9, assessing the reporting of numerical outcome estimates and their precision (e.g., confidence intervals).

## DISCUSSION

Based on the CONSORT 2025 guidelines, the areas that were generally underreported can be discussed as follows. First, there was insufficient reporting on Open Science, including trial registration, the protocol, and statistical analysis plan, and data sharing. These elements were newly added in the 2025 revision of CONSORT to reflect the global trend toward open science, emphasizing openness and accountability in research through registration, data and code sharing, and conflict of interest reporting [6]. When examining five-year reporting trends, no studies published between 2011 and 2020 reported items related to Open Science, whereas 75% of those published between 2021 and 2024 did. Considering that the revision was made in 2025, it is evident that reporting on these elements had begun to emerge gradually even before the formal update, reflecting the evolving trend of the times.

Regarding the public disclosure of protocols and statistical analysis plans, discrepancies were identified between the registered protocol and the full report, particularly concerning primary outcome variables and sample sizes [17]. This finding suggests that making statistical analysis plans (SAP) publicly available could play a crucial role in preventing discrepancies between research design and reporting. The insufficient reporting of data sharing is consistent with previous studies [18], which have shown that although many journals require a data sharing statement in their submission guidelines, published articles still often lack this information. Data sharing not only promotes transparency but also enhances the reproducibility of research findings, enables secondary analyses and meta-analyses, and contributes to public health and scientific progress. Therefore, it is essential to ensure that data sharing involves not only a formal declaration but also actual execution and reporting [18].

To ensure transparency and validity in research, it is particularly important to emphasize the role of trial registration, the public disclosure of protocols and statistical analysis plans, and data sharing. Recent studies have shown that when these elements are insufficient, they can lead to bias in outcome reporting and potentially distort the estimates for adverse events (harms) or primary outcomes [19]. CONSORT 2025 emphasizes registration, protocols, SAP, and data sharing as separate sections. In the case of JKBNS, the CRIS system is primarily used, and all three relevant articles were documented in the main text rather than in the abstracts. Specifically, these items were reported in the Ethical Considerations section of the main text [20].

This study highlights the need for researchers to place greater emphasis on reporting within the open science framework. By faithfully applying these standards, including the transparent reporting of registered protocols, analysis plans, and data availability, future research will be able to enhance both the credibility and impact of its findings.

On the other hand, there was insufficient reporting in the Methods section regarding changes to the trial protocol, harm definition and assessment, and interim analyses and stopping guidelines. First, reporting changes to the trial protocol was insufficient, limiting transparency into procedural modifications or alterations to analysis plans that occurred during the study. Second, reporting related to harm definition and assessment was inadequate, making it difficult to systematically evaluate adverse events or unintended effects resulting from interventions, thereby posing challenges for readers attempting to replicate or interpret the findings. In accordance with CONSORT Harms and the 2025 CONSORT update, nursing intervention studies should explicitly define, systematically monitor, and report adverse events. Although nursing interventions are generally considered low-risk, specific outcomes—such as increased patient fatigue, procedural discomfort, transient anxiety, or minor physiological changes—should be prospectively identified and documented using standardized criteria. Transparent reporting of such events not only strengthens methodological rigor but also ensures ethical accountability and reproducibility in nursing research. Lastly, interim analyses and stopping guidelines were not reported, which meant there was no clear evidence provided regarding safety reviews through interim analyses or the possibility of early trial termination. Such limitations in reporting may weaken the credibility and reproducibility of research [21]. Therefore, future studies should provide detailed descriptions of these elements in accordance with the CONSORT 2025 guidelines.

Reporting related to randomization and blinding was found to be insufficient. Specifically, while descriptions of sequence generation in the randomization process were provided, reporting on allocation concealment —known to significantly affect a study's internal validity— was lacking. This may be due to the nature of nursing research, in which researchers often act as facilitators, making it challenging to maintain allocation concealment. Similarly, given the nature of nursing interventions, it may have been challenging to implement blinding effectively. In this study, the reporting of both the randomization procedure and the blinding methods was insufficiently detailed. Without a clear explanation of the randomization process and allocation concealment, it is difficult to completely rule out selection bias. Likewise, inadequate reporting on whether participants, researchers, and outcome assessors were blinded — as well as the level of blinding (single-blind, double-blind, assessor-blind, etc.) — can reduce the objectivity of outcome measurement and introduce performance and detection bias when interpreting the results. The findings of this study revealed that the reporting rates for “Who was blinded” and “How blinding was achieved” were generally low. In this review, blinding was explicitly reported only in studies in which both participants and data collectors were blinded [22] and in which only participants were blinded [23,24]. This low rate of reporting is likely attributable to the fact that most nursing interventions are non-pharmacological, making it inherently difficult to implement blinding procedures. The CONSORT 2025 guidelines emphasize the need to provide detailed descriptions of randomization and blinding procedures [6]. Previous studies have repeatedly pointed out that a lack of such information undermines the credibility and reproducibility of clinical trial findings [16,25]. Therefore, in situations where blinding is challenging due to the nature of nursing interventions, strategies to minimize bias—such as separating participants from outcome assessors—should be implemented. Future research should also systematically report on the specific methods used for randomization and blinding to ensure transparency and rigor. Moreover, nursing research often operates with limited funding resources and is typically conducted on a small scale. As a result, researchers themselves often perform multiple roles—including randomization, intervention delivery, and data collection—rather than assigning these tasks to separate personnel. Such structural constraints may further hinder the feasibility of implementing blinding procedures. Therefore, future nursing studies should explore practical strategies to strengthen blinding procedures during the design phase and advocate for institutional and policy-level efforts to expand funding support. Increased funding for nursing research is essential to enhance the methodological rigor and credibility of intervention studies in this field.

In the Statistical Methods section, insufficient reporting was provided on missing data handling, additional analyses, and participant flow. Similarly, in the Reason for Trial Ending section, reporting was limited to the reasons for trial ending, harms, and ancillary analyses. Only one study [26] reported an incident related to harm, such as a needlestick injury. Apart from this, no other studies provided information regarding harm. This may be due to the nature of the interventions included in nursing research, which are generally non-invasive, educational, or non-pharmacological. Consequently, the absence of reported harm likely reflects the lack of adverse events rather than a failure to report them. However, it is essential to note that any adverse event arising from an intervention should be explicitly reported in accordance with the CONSORT Harms extension.

Specifically, there was a lack of detailed descriptions of missing-data handling, making it difficult for readers to clearly understand the potential bias introduced by the method used to address missing data. Reporting on additional analyses (e.g., subgroup analyses, sensitivity analyses) was also limited. Furthermore, the reporting of participant flow was insufficient, making it difficult to transparently identify participant enrollment, dropouts, and inclusion in the final analysis. In addition, there were notable limitations in the Reason for Trial Ending section. Without a clear explanation of why the trial was terminated, it becomes difficult to assess the study's legitimacy and validity fully. The incomplete reporting of harms reduces the reliability of evaluating adverse events and safety outcomes. Moreover, the lack of reporting on ancillary analyses limits the potential for additional interpretation of the study’s findings. Given the increasing emphasis on transparency, safety, and reproducibility in research [27], improvements in the reporting of statistical methods are needed to strengthen the overall quality and credibility of studies.

In this study, the number of published RCTs was relatively low in 2016, 2020, 2022, and 2023. This finding is consistent with previous reviews conducted across multiple countries that reported substantial enrollment delays, operational gaps, and difficulties in participant recruitment during this period [28]. The overall social context of the coronavirus disease 2019 pandemic, characterized by restrictions on face-to-face interactions and disruptions in healthcare operations, likely contributed to these challenges. Given that experimental studies require rigorous control of interventions and study conditions, it is reasonable to infer that conducting RCTs during this time was particularly constrained by pandemic-related limitations.

Reflecting differences in reporting feasibility across study designs, this study found that crossover trials tended to yield lower compliance scores when assessed against RCT reporting guidelines. This pattern may reflect the inherent methodological challenges of crossover designs, in which complete blinding of participants and data collectors is often unfeasible. Moreover, essential methodological details—such as blinding procedures or allocation concealment—were frequently underreported or ambiguously stated, possibly because they were regarded as self-evident within the study design. Although specific items may have been deemed “non-applicable,” we adhered strictly to the CONSORT-based evaluation criteria and assigned a score of 0 when reporting was insufficient. Therefore, lower compliance scores in crossover trials should be interpreted with consideration of the study design’s inherent limitations, underscoring the need for transparent, detailed reporting in future research to enhance methodological rigor and interpretability.

In this study, we compared (mapped) the items of the CONSORT 2025 reporting guideline with those of three major quality assessment tools: RoB 2, SIGN, and JBI Critical Appraisal Criteria. The characteristics of each tool are as follows: RoB 2 is a risk of bias assessment tool for RCTs developed by Cochrane. It evaluates the systematic risk of bias in five key domains: randomization process, implementation of interventions, measurement of outcomes, and selective reporting [7]. It categorizes each domain as “Low risk,” “Some concerns,” or “High risk,” providing an overall judgment of study reliability. The SIGN checklist is applicable to a variety of study designs, including RCTs, cohort studies, and case-control studies [8]. It evaluates factors such as the clarity of the research question, randomization and blinding, handling of missing data, and appropriateness of analysis. The quality of studies is graded as ++ (High quality), + (Acceptable), or – (Low quality). The JBI Critical Appraisal Tools, developed by the JBI in Australia, provide tailored checklists for nearly all study designs, including RCTs, cohort studies, single-case studies, and qualitative research [9]. Each item is evaluated as Yes, No, Unclear, or Not Applicable. A key strength of the JBI tools is their ability to assess the quality of evidence across a wide range of healthcare research, including qualitative and mixed methods studies.

In this study, a comparison was made between the CONSORT 2025 reporting guideline and the key items of major quality assessment tools (RoB 2, SIGN, and JBI Critical Appraisal Criteria). The results showed that these quality assessment tools primarily focus on evaluating the internal validity and reliability of studies, particularly by examining factors that may threaten validity. In this respect, their purpose aligns with that of CONSORT. In the Methods domain, most items matched closely between CONSORT and the quality assessment tools, while in the Results domain, partial alignment was also observed.

However, certain technical aspects, such as risk of bias, emphasized in RoB 2 and JBI [7,29], were less well addressed in CONSORT, which overall tended to focus more on methodological reporting. These findings indicate that while CONSORT plays an essential role as a guideline for enhancing the transparency of clinical trial reporting, its focus differs from that of tools designed to evaluate research quality. Therefore, for a comprehensive interpretation of clinical trial reporting and evaluation, it is necessary to use CONSORT alongside quality assessment tools such as RoB 2, SIGN, and JBI.

This integrated approach is meaningful in that it goes beyond simply assessing RCT bias. A multilayered evaluation of study quality based on research design provides a stronger foundation for systematically interpreting the credibility and applicability of research findings.

## CONCLUSION

This study evaluated the completeness of reporting of RCTs published in the JKBNS between 2011 and 2024, using the CONSORT 2025 guidelines. The results showed that most RCTs adopted a standard parallel-group design, and a significant portion of the intervention studies focused on non-pharmacological interventions, particularly aromatherapy. While some CONSORT checklist items were consistently reported, essential methodological elements — such as protocol modifications, interim analyses, and handling of missing data — were notably underreported. In addition, 14 CONSORT items were found to conceptually align with the methodological quality domains of RoB 2.0, SIGN, and JBI critical appraisal tools.

These findings suggest that the overall reporting quality of RCTs published in JKBNS is moderate. Moving forward, nursing researchers should more comprehensively and systematically adhere to standardized reporting guidelines, such as the CONSORT checklist, when designing and reporting RCTs. By doing so, it will be possible to enhance the transparency and credibility of research and strengthen its applicability in clinical practice.

## Figures and Tables

**Table 1.** Characteristics of Randomized Controlled Trials Published in JKBNS (2011~2024)

Study ID	Author (year)	Study design	Randomization methods	Participants	Type of intervention	Duration of intervention	Session of intervention	Time/session	Control group intervention	Outcomes
1	Park et al. (2011) [A1]	RCT crossover	Not reported	18 college students (E^a^:18, E^b^:18, E^c^:18, C:18)	Hand-drying methods after handwashing (E1: paper towel, E2: hand dryer while rubbing the hands, E3: hand dryer without rubbing the hands)	4 days	4	20 seconds~2 minutes	Natural hand drying for 7~8 minutes	Residual bacteria count after hand washing
2	Won & Chae (2011) [A2]	RCT	Lottery methods	42 older adults with degenerative knee osteoarthritis (E:21, C:21)	Aromatherapy massage	4 weeks	8	20 minutes	No	Pain (NRS), sleep disturbance (Korean Sleep Scale), and stride length
3	Seol & Jung (2011) [A3]	RCT	Not reported	52 surgery patients for lumbar spinal stenosis (E:26, C:26)	Aromatherapy oil inhalation (Bergamot essential oil-inhalation)	1 day	1	20 minutes	Almond oil-inhalation	Pain (VAS), blood pressure, heart rate, and respiratory rate
4	Choi & Lee (2012) [A4]	RCT	Random number table	36 patients with essential hypertension (E:20, C:16)	Aromatherapy oil inhalation (blended oils of Lavender, Majoram, and Ylang Ylang in a ratio of 4:3:3)	2 weeks	42	3 minutes	No	Blood pressure, pulse, sleep disturbance, stress, and anxiety
5	Synn & Choe (2012) [A5]	RCT crossover	Order of study registration using a random number table.	21 patients who were being weaned from mechanical ventilators (E^a^: 21, E^b^:21, C:21)	Music therapy (E1: western music, E2: Korean traditional music)	1 day	1	30 minutes	Rest	Physiological indices, anxiety, and dyspnea
6	Kim et al. (2012) [A6]	RCT	R 2.14.2 (R core team)	62 nursing students (E:31, C:31)	Phytoncide aromatherapy	2 weeks	1	2 weeks	Normal saline	Stress, symptoms of stress (Symptoms of Stress Scale), and heart rate variability
7	Lee & Yoon (2012) [A7]	RCT	Table of random numbers	132 patients undergoing inhalation general anesthesia (E:66, C:66)	0.5 mg/kg lidocaine	1 day	1	Not reported	0.5 mg/kg saline	Fentanyl-induced cough, mean arterial pressure, heart rate, oxygen saturation, and dizziness
8	Cho & Cho (2014) [A8]	RCT	Coin tossing method	81 nursing students (E:40, C:41)	Aromatherapy oral gargling (blended oils of peppermint, lemon, tea tree in a ratio of 2:2:1)	1 day	1	15 seconds	0.9% normal saline	Xerostomia (VAS), halitosis, and salivary pH
9	Joung et al. (2014) [A9]	RCT	The order in which participants enrolled	90 college students (E^a^: 30, E^b^:30, C:30)	Hand washing (E1: antibacterial soap, E2: plain soap+ education)	1 day	1	30 seconds	Plain soap	The removal rate of *Escherichia coli* and *Staphylococcus aureus*
10	Kim & Jo (2014) [A10]	RCT	Lottery methods	60 patients who underwent gynecological surgery (E:30, C:30)	Nurse presence (NP) program	1 day	1	20~30 minutes	No	Anxiety and physiological indicators
11	Sin et al. (2015) [A11]	RCT	Not reported	68 older Korean immigrants (E:44, C:22)	Walking program (pedometer, goal setting (10,000 steps per day), and walking with a buddy)	12 weeks	6	Not reported	Usual walking	walking behavior (pedometer steps count), stress (cortisol), depressive symptoms (CESD-10), and cardiovascular disease biomarkers (hs-CRP and fibrinogen
12	Kim et al. (2015) [A12]	RCT	Lottery methods	49 female college students (E^a^: 12, E^b^:18, C: 19)	Auricular acupressure (E1: on the first or second day of their menstrual period, E2: once a week for a month)	E1: 1 day, E2: 4 weeks	E1: 1, E2: 4	E1, 2: 10~15 minutes	No	Menstrual pain, difficulties in daily life, negative feelings, and autonomic nervous responses
13	Seo & Yoon (2015) [A13]	RCT	Computer-generated randomization method	69 patients who underwent laparoscopic colectomy under general anesthesia (E^a^:23, E^b^:23, C:23)	Music and noise blocking (E1: music therapy, E2: noise blocking)	1 day	1	from admission to the recovery room until discharge	No	Postoperative pain, length of stay in the post-anesthetic care unit, and satisfaction
14	Park & Yoon (2017) [A14]	RCT	Excel’s built-in randomization functions	74 patients who underwent laparoscopic colectomy under general anesthesia (E:36, C:38)	37˚C carbon dioxide pneumoperitoneum	1 day	1	until the end of the surgical procedure	21˚C CO_2_ pneumoperitoneum	Core body temperature, systolic blood pressure, heart rate, and acid-base balance
15	Lee et al. (2018) [A15]	RCT	Randomizer research program (http://www.randomizer.org)	91 patients in MICU (E:47, C:44)	Daily chlorhexidine bathing (2% CHG bathing)	Not reported	1	10 minutes	traditional bath	Acquisition of multidrug-resistant organisms and healthcare-associated infection
16	Park et al. (2019) [A16]	RCT	Computer-based random number generation software	52 gastrointestinal cancer survivors (E:21, C:31)	Laughter therapy	8 weeks	8	60 minutes	No	Depression and anxiety (the Hospital Anxiety and Depression Scale), fatigue (the Fatigue Severity Scale), and quality of sleep (the Verran & Snyder-Halpern Sleep Scale)
17	Kang & Chae (2019) [A17]	RCT crossover	Random allocation in the desired sequence	18 patients with chronic renal failure receiving hemodialysis treatment (E^a^:18, C:18)	Skin stimulation method	2 weeks	2	Not reported	Topical anesthetic cream	Pain (VAS), stress, and heart rate variability
18	Lee (2019) [A18]	RCT	Computer-generated random numbers via the Research Randomizer.	70 nursing students (E:35, C:35)	Finger guard	1 day	1	Not reported	No	Sharp injuries, anxiety (VAS), and user satisfaction (K-QUEST2.0 (Quebec User Evaluation of Satisfaction with Assistive Technology)
19	Kim et al. (2021) [A19]	RCT	Randomization function using Microsoft Excel	36 participants undergoing chemotherapy (E^a^:12, E^b^:12, C:12)	Aromatherapy oil inhalation (E1: 5% clary sage oil, E2: 5% linalyl acetate)	1 day	1	20 minutes	Almond oil inhalation	State-trait anxiety inventory (STAI), Stress rating scale, anxiety-visual analog scale (Anxiety-VAS), stress-visual analog scale (Stress-VAS), blood pressure, and heart rate were
20	Her & Hur (2024) [A20]	RCT	Excel's random number generation method	57 participants diagnosed with allergic rhinitis (E:28, C:29)	Mask applied aromatherapy (eucalyptus, lemon, roman chamomile, and peppermint in a ratio of 2:2:1:0.5)	2 weeks	28	Not reported	No	Allergic rhinitis symptoms (VAS, TNSS), fatigue (Fatigue Self-awareness Survey), and quality of life (ARSQOL)
21	Lee & Hur (2024) [A21]	RCT	Excel randomization functions	59 hospitalized adults who had an 18-gauge Angio catheter inserted for surgery (E^a^:20, E^b^:20, C:19)	Thermoelectric tourniquet (E1: 40°C-45°C thermotherapy, E2: 0°C-10°C cryotherapy)	1 day	1	10~30 seconds	M-TEE tourniquet without temperature control	Pain, regional blood flow, and satisfaction
22	Lee et al. (2024) [A22]	RCT	Computer-generated random numbers	78 shift nurses (E^a^:26, E^b^: 26, C:26)	Music and aromatherapy (E1: music therapy, E2: aromatherapy necklace (Lavender, Ylang Ylang, and Lemon in a 5:5:1 ratio)	1 week	E1: 21, E2: continuous	E1: 30 minutes, E2: continuous	Usual daily activities	Stress (perceived stress and stress index), quality of life (WHOQOL-BREF), and happiness (The Oxford Happiness Questionnaire)

Superscripts a, b, and c were divided by intervention. JKBNS = *Journal of Korean Biological Nursing Science*; RCT = Randomized controlled trial; E = Experimental group; C = Control group; CHG = Chlorhexidine gluconate; NRS = Numeric rating scale; VAS = Visual analog scale; CESD = Center for Epidemiologic Studies Depression Scale; hs-CRP = High-sensitivity C-reactive protein; TNSS = Total Nasal Symptom Score; ARSQOL = Allergic Rhinitis‑Specific Quality of Life; WHOQOL-BREF = World Health Organization Quality of Life – brief form.

**Table 2.** Evaluation of Reporting Quality of Randomized Controlled Trials Published in JKBNS (2011~2024) Based on the CONSORT 2025 Guidelines

Domains	Items	1	2	3	4	5	6	7	8	9	10	11	12	13	14	15	16	17	18	19	20	21	22	Sum
Title and abstract	1a. Identification as a randomized trial in the title	0	0	0	0	0	0	0	0	0	0	0	0	0	1	0	1	0	0	1	1	1	1	6
	1b. Structured summary of the trial design, methods, results, and conclusions	1	1	1	1	1	1	1	1	1	1	1	1	1	1	1	1	1	1	1	1	1	1	22
Open science	2. Trial registration	0	0	0	0	0	0	0	0	0	0	0	0	0	0	0	0	0	0	0	1	1	1	3
	3. Protocol and statistical analysis plan	0	0	0	0	0	0	0	0	0	0	0	0	0	0	0	0	0	0	0	1	1	1	3
	4. Data sharing	0	0	0	0	0	0	0	0	0	0	0	0	0	0	0	0	0	0	0	1	1	1	3
Funding and conflicts	5a. Sources of funding and role of funders	0	0	1	0	0	0	0	0	0	0	0	1	0	0	0	1	0	1	1	1	1	1	8
	5b. Conflicts of interest	0	0	0	0	0	0	0	0	0	0	0	0	0	0	1	1	1	1	1	1	1	1	8
Introduction	6. Scientific background and rationale	1	1	1	1	1	1	1	1	1	1	1	1	1	1	1	1	1	1	1	1	1	1	22
	7. Specific objectives	1	1	1	1	1	1	1	1	1	1	1	1	1	1	1	1	1	1	1	1	1	1	22
Methods	8. Patient and public involvement	0	0	0	1	0	0	0	0	0	0	0	0	1	0	0	1	0	1	1	1	1	1	8
	9. Trial design	1	1	1	1	1	1	1	1	1	1	1	1	1	1	1	1	1	1	1	1	1	1	22
	10. Changes to trial protocol	0	0	0	0	0	0	0	0	0	0	0	0	0	0	0	0	0	0	0	0	0	0	0
	11. Trial setting	1	1	1	1	1	1	1	1	1	1	1	1	1	1	1	1	1	1	1	1	1	1	22
	12a. Eligibility criteria for participants	1	1	1	1	1	1	1	1	1	1	1	1	1	1	1	1	1	1	1	1	1	1	22
	12b. Eligibility criteria for sites/providers	1	1	1	1	1	1	1	1	1	1	1	1	1	1	1	1	1	1	1	1	1	1	22
	13. Intervention and comparator details	1	1	1	1	1	1	1	1	1	1	1	1	1	1	1	1	1	1	1	1	1	1	22
	14. Prespecified outcomes	1	1	1	1	1	1	1	1	1	1	1	1	1	1	1	1	1	1	1	1	1	1	22
	15. Harms definition and assessment	0	0	0	0	0	0	1	1	0	0	0	1	0	0	1	0	0	1	1	1	1	1	9
	16a. Sample size determination	0	1	1	1	1	1	1	1	1	1	1	1	1	1	1	1	1	1	1	1	1	1	21
	16b. Interim analyses and stopping guidelines	0	0	0	0	0	0	0	0	0	0	0	0	0	0	0	0	0	0	0	0	0	0	0
Randomization	17a. Sequence generation	0	1	0	1	1	1	1	1	1	1	1	1	1	1	1	1	1	1	1	1	1	1	20
	17b. Type of randomization and restriction	0	1	0	1	1	1	1	1	1	0	0	0	0	0	0	1	0	0	1	0	1	1	11
	18. Allocation concealment mechanism	0	0	0	0	0	1	1	1	0	0	0	0	0	1	0	1	0	0	1	0	1	1	8
	19. Implementation	1	1	1	1	1	1	1	1	1	1	1	1	1	1	1	1	1	1	1	1	1	1	22
Blinding	20a. Who was blinded	0	0	0	0	0	1	0	0	0	0	0	0	0	1	0	1	0	0	0	0	0	0	3
	20b. How blinding achieved	0	0	0	0	0	0	0	1	0	0	0	0	0	1	0	0	0	0	1	1	0	0	4
Statistical methods	21a. Statistical methods	0	1	1	1	1	1	1	1	0	1	1	1	1	1	1	1	1	1	1	1	1	1	20
	21b. Definition of analysis population	1	1	1	1	1	1	1	1	1	1	1	1	1	1	1	1	1	1	1	1	1	1	22
	21c. Missing data handling	0	0	0	0	0	0	0	0	0	0	0	0	0	0	0	0	0	0	0	0	0	0	0
	21d. Additional analyses	0	0	0	0	0	0	0	0	1	0	0	0	0	0	0	0	0	0	0	0	0	0	1
Results	22a. Participant flow	0	0	0	0	0	0	0	0	0	0	0	1	1	1	1	1	1	0	0	0	0	0	6
	22b. Losses and exclusions	1	1	1	1	1	1	1	1	1	1	1	1	1	1	1	1	1	1	1	1	1	1	22
	23a. Recruitment and follow-up dates	1	1	1	1	1	1	1	1	1	1	1	1	1	1	1	1	1	1	1	1	1	1	22
	23b. Reason for trial ending	0	0	0	0	0	0	0	0	0	0	0	0	0	0	0	0	0	0	0	0	0	0	0
	24a. Intervention delivery	1	1	1	1	1	1	1	1	1	1	1	1	1	1	1	1	1	1	1	1	1	1	22
	24b. Concomitant care	0	0	1	0	0	0	1	1	1	0	0	1	1	1	1	0	1	0	1	0	0	0	10
	25. Baseline data	1	1	1	1	1	1	1	1	1	1	1	1	1	1	1	1	1	0	0	0	0	0	17
	26. Outcomes and estimation	1	1	1	1	1	1	1	1	1	1	1	1	1	1	1	1	1	1	1	1	1	1	22
	27. Harms	0	0	0	0	0	0	0	0	0	0	0	0	0	0	0	0	0	1	0	0	0	0	1
	28. Ancillary analyses	1	0	0	0	0	0	0	0	0	0	0	0	0	0	0	0	0	0	0	0	0	0	1
Discussion	29. Interpretation	1	1	1	1	1	1	1	1	1	1	1	1	1	1	1	1	1	1	1	1	1	1	22
	30. Limitations	1	1	1	1	1	1	1	1	1	1	1	1	1	1	1	1	1	1	1	1	1	1	22
Total CONSORT score per study		19	22	22	23	22	24	25	26	23	21	21	25	24	27	25	29	24	25	29	29	30	30	24.77

JKBNS = *Journal of Korean Biological Nursing Science*; CONSORT = Consolidated Standards of Reporting Trials.

**Table 3.** Trend Analysis Across Combined Years of Studies Published in JKBNS (2011~2024) Based on CONSORT 2025 Guidelines

Domains	Items	2011~2015 (n = 13)	2016~2020 (n = 5)	2021~2024 (n = 4)	Total (n = 22)
Title and abstract	1a. Identification as a randomized trial in the title	0 (0.0)	2 (40.0)	4 (100.0)	6 (27.3)
	1b. Structured summary of the trial design, methods, results, and conclusions	13 (100.0)	5 (100.0)	4 (100.0)	22 (100.0)
Open science	2. Trial registration	0 (0.0)	0 (0.0)	3 (75.0)	3 (13.6)
	3. Protocol and statistical analysis plan	0 (0.0)	0 (0.0)	3 (75.0)	3 (13.6)
	4. Data sharing	0 (0.0)	0 (0.0)	3 (75.0)	3 (13.6)
Funding and conflicts	5a. Sources of funding and role of funders	2 (15.4)	2 (40.0)	4 (100.0)	8 (36.4)
	5b. Conflicts of interest	0 (0.0)	4 (80.0)	4 (100.0)	8 (36.4)
Introduction	6. Scientific background and rationale	13 (100.0)	5 (100.0)	4 (100.0)	22 (100.0)
	7. Specific objectives	13 (100.0)	5 (100.0)	4 (100.0)	22 (100.0)
Methods	8. Patient and public involvement	2 (15.4)	2 (40.0)	4 (100.0)	8 (36.4)
	9. Trial design	13 (100.0)	5 (100.0)	4 (100.0)	22 (100.0)
	10. Changes to trial protocol	0 (0.0)	0 (0.0)	0 (0.0)	0 (0.0)
	11. Trial setting	13 (100.0)	5 (100.0)	4 (100.0)	22 (100.0)
	12a. Eligibility criteria for participants	13 (100.0)	5 (100.0)	4 (100.0)	22 (100.0)
	12b. Eligibility criteria for sites/providers	13 (100.0)	5 (100.0)	4 (100.0)	22 (100.0)
	13. Intervention and comparator details	13 (100.0)	5 (100.0)	4 (100.0)	22 (100.0)
	14. Prespecified outcomes	13 (100.0)	5 (100.0)	4 (100.0)	22 (100.0)
	15. Harms definition and assessment	3 (23.1)	2 (40.0)	4 (100.0)	9 (40.9)
	16a. Sample size determination	12 (92.3)	5 (100.0)	4 (100.0)	21 (95.5)
	16b. Interim analyses and stopping guidelines	0 (0.0)	0 (0.0)	0 (0.0)	0 (0.0)
Randomization	17a. Sequence generation	11 (84.6)	5 (100.0)	4 (100.0)	20 (90.9)
	17b. Type of randomization and restriction	7 (53.8)	1 (20.0)	3 (75.0)	11 (50.0)
	18. Allocation concealment mechanism	3 (23.1)	2 (40.0)	3 (75.0)	8 (36.4)
	19. Implementation	13 (100.0)	5 (100.0)	4 (100.0)	22 (100.0)
Blinding	20a. Who was blinded	1 (7.7)	2 (40.0)	0 (0.0)	3 (13.6)
	20b. How blinding achieved	1 (7.7)	1 (20.0)	2 (50.0)	4 (18.2)
Statistical methods	21a. Statistical methods	11 (84.6)	5 (100.0)	4 (100.0)	20 (90.9)
	21b. Definition of analysis population	13 (100.0)	5 (100.0)	4 (100.0)	22 (100.0)
	21c. Missing data handling	0 (0.0)	0 (0.0)	0 (0.0)	0 (0.0)
	21d. Additional analyses	1 (7.7)	0 (0.0)	0 (0.0)	1 (4.5)
Results	22a. Participant flow	2 (15.4)	4 (80.0)	0 (0.0)	6 (27.3)
	22b. Losses and exclusions	13 (100.0)	5 (100.0)	4 (100.0)	22 (100.0)
	23a. Recruitment and follow-up dates	13 (100.0)	5 (100.0)	4 (100.0)	22 (100.0)
	23b. Reason for trial ending	0 (0.0)	0 (0.0)	0 (0.0)	0 (0.0)
	24a. Intervention delivery	13 (100.0)	5 (100.0)	4 (100.0)	22 (100.0)
	24b. Concomitant care	6 (46.2)	3 (60.0)	1 (25.0)	10 (45.5)
	25. Baseline data	13 (100.0)	4 (80.0)	0 (0.0)	17 (77.3)
	26. Outcomes and estimation	13 (100.0)	5 (100.0)	4 (100.0)	22 (100.0)
	27. Harms	0 (0.0)	1 (20.0)	0 (0.0)	1 (4.5)
	28. Ancillary analyses	1 (7.7)	0 (0.0)	0 (0.0)	1 (4.5)
Discussion	29. Interpretation	13 (100.0)	5 (100.0)	4 (100.0)	22 (100.0)
	30. Limitations	13 (100.0)	5 (100.0)	4 (100.0)	22 (100.0)

Values are presented as n (%). JKBNS = *Journal of Korean Biological Nursing Science*; CONSORT = Consolidated Standards of Reporting Trials.

**Table 4.** Mapping of CONSORT 2025 Items to RoB 2, SIGN, and JBI Critical Appraisal Criteria

Domains	CONSORT 2025 items	RoB 2.0	SIGN	JBI
Title and abstract	1a. Identification as a randomized trial in the title			
	1b. Structured summary of the trial design, methods, results, and conclusions			
Open science	2. Trial registration	5.1		
	3. Protocol and statistical analysis plan	5.1		12
	4. Data sharing			
Funding and conflicts	5a. Sources of funding and role of funders			
	5b. Conflicts of interest			
Introduction	6. Scientific background and rationale		1.1	
	7. Specific objectives		1.1	
Methods	8. Patient and public involvement			
	9. Trial design			13
	10. Changes to trial protocol	5.1		13
	11. Trial setting			
	12a. Eligibility criteria for participants			
	12b. Eligibility criteria for sites/providers			
	13. Intervention and comparator details		1.6	6
	14. Prespecified outcomes	4.1, 4.2, 5.2, 5.3	1.7	8, 9
	15. Harms definition and assessment			
	16a. Sample size determination			
	16b. Interim analyses and stopping guidelines			
Randomization	17a. Sequence generation	1.1	1.2, 2.1	1
	17b. Type of randomization and restriction	1.1	1.2, 2.1	1
	18. Allocation concealment mechanism	1.2	1.3, 2.1	2
	19. Implementation	1.2	2.1	
Blinding	20a. Who was blinded	2.1, 2.2, 4.3	1.4, 2.1	4,5,7
	20b. How blinding achieved	2.1, 2.2	1.4, 2.1	4,5,7
Statistical methods	21a. Statistical methods	2.6, 2.7		12
	21b. Definition of analysis population	2.6	1.9	11
	21c. Missing data handling	3.1-3.4	2.1	10
	21d. Additional analyses	5.2, 5.3		
Results	22a. Participant flow	3.1	2.1	10
	22b. Losses and exclusions	3.1~3.4	1.8, 2.1	10
	23a. Recruitment and follow-up dates			
	23b. Reason for trial ending			
	24a. Intervention delivery	2.3~2.5	1.6	6
	24b. Concomitant care	2.3~2.5	1.6	6
	25. Baseline data	1.3	1.5, 2.1	3
	26. Outcomes and estimation	4.1~4.5	2.1	8,9
	27. Harms			
	28. Ancillary analyses	5.2, 5.3	1.10	
Discussion	29. Interpretation		2.2, 2.4	
	30. Limitations		2.1, 2.2, 2.3, 2.4	

CONSORT = Consolidated Standards of Reporting Trials; RoB 2.0 = Risk of Bias 2.0; SIGN = Scottish Intercollegiate Guidelines Network; JBI = Joanna Briggs Institute.

## Data Availability

The data that support the findings of this study are available from the corresponding author upon reasonable request.

## Appendix 1. Studies included in the systematic review and meta-analysis

**A1.** Park JS, Kim DB, Min HG. Comparison of desiccation methods after hand washing for removing bacteria. Journal of Korean Biological Nursing Science. 2011;13(1):8-15.

**A2.** Won SJ, Chae YR. The effects of aromatherapy massage on pain, sleep, and stride length in the elderly with knee osteoarthritis. Journal of Korean Biological Nursing Science. 2011;13(2):142-148.

**A3.** Seol GH, Jung MH. Effect of bergamot essential oil inhalation on chronic pain after surgery for lumbar spinal stenosis. Journal of Korean Biological Nursing Science. 2011;13(2):156-163.

**A4.** Choi EM, Lee KS. Effects of aroma inhalation on blood pressure, pulse rate, sleep, stress, and anxiety in patients with essential hypertension. Journal of Korean Biological Nursing Science. 2012;14(1):41-48. https://doi.org/10.7586/jkbns.2012.14.1.41

**A5.** Synn A, Choe MA. Effect of music therapy on the physiological index, anxiety, and dyspnea of patients with mechanical ventilator weaning. Journal of Korean Biological Nursing Science. 2012;14(1):57-65. https://doi.org/10.7586/jkbns.2012.14.1.57

**A6.** Kim CG, Cho MK, Kim JI. Effects of phytoncide aromatherapy on stress, symptoms of stress, and heart rate variability among nursing students. Journal of Korean Biological Nursing Science. 2012;14(4):249-257. https://doi.org/10.7586/jkbns.2012.14.4.249

**A7.** Lee KY, Yoon H. The effect of low dose lidocaine on fentanyl-induced cough, mean arterial pressure, heart rate, oxygen saturation, and dizziness in inhalation anesthesia. Journal of Korean Biological Nursing Science. 2012;14(4):275-281. https://doi.org/10.7586/jkbns.2012.14.4.275

**A8.** Cho MK, Cho YH. Comparisons of the effects of A-solution and 0.9% normal saline oral gargling on xerostomia, halitosis, and salivary pH in nursing students. Journal of Korean Biological Nursing Science. 2014;16(2):141-149. https://doi.org/10.7586/jkbns.2014.16.2.141

**A9.** Joung HY, Choi Y, Hyun HJ, Kim JH, Yoon SJ, Lee G. Comparison of bacterial cultivation results before and after hand washing from a college student in Gangwon Province, Korea: using plain and antibacterial soap. Journal of Korean Biological Nursing Science. 2014;16(3):157-163. https://doi.org/10.7586/jkbns.2014.16.3.157

**A10.** Kim YJ, Jo KH. Effects of nurse presence program on anxiety and physiological indicators in patients with gynecological surgery. Journal of Korean Biological Nursing Science. 2014;16(4):326-333. https://doi.org/10.7586/jkbns.2014.16.4.326

**A11.** Sin MK, Ibarra B, Tae T, Murphy PJM. Effect of a randomized controlled trial walking program on walking, stress, depressive symptoms, and cardiovascular biomarkers in elderly Korean immigrants. Journal of Korean Biological Nursing Science. 2015;17(2):89-96. https://doi.org/10.7586/jkbns.2015.17.2.89

**A12.** Kim NY, Kim MA, Choi SE. Effects of auricular acupressure on menstrual pain, difficulties in daily life, negative feelings, and autonomic nervous responses in female college students. Journal of Korean Biological Nursing Science. 2015;17(2):159-168. https://doi.org/10.7586/jkbns.2015.17.2.159

**A13.** Seo E, Yoon H. Comparison of the effect of music and noise blocking on postoperative pain, length of stay at post anesthetic care unit, and satisfaction after a laparoscopic colectomy. Journal of Korean Biological Nursing Science. 2015;17(4):315-323. https://doi.org/10.7586/jkbns.2015.17.4.315

**A14.** Park JI, Yoon H. Effects of 37˚C carbon dioxide pneumoperitoneum on core body temperature, systolic blood pressure, heart rate, and acid-base balance: a randomized double-blind controlled trial. Journal of Korean Biological Nursing Science. 2017;19(2):76-85. https://doi.org/10.7586/jkbns.2017.19.2.76

**A15.** Lee JY, Jeong JS, Kim MY, Park SH, Hwang YH. Effects of daily chlorhexidine bathing on the acquisition of multidrug-resistant organisms and healthcare-associated infection in an intensive care unit. Journal of Korean Biological Nursing Science. 2018;20(1):38-46. https://doi.org/10.7586/jkbns.2018.20.1.38

**A16.** Park SY, Lee YS, Chung HH, Choi-Kwon S. Effects of laughter therapy on depression, anxiety, fatigue, and quality of sleep in gastrointestinal cancer patients post-treatment: a randomized controlled trial. Journal of Korean Biological Nursing Science. 2019;21(3):188-198. https://doi.org/10.7586/jkbns.2019.21.3.188

**A17.** Kang HY, Chae YR. Comparison of skin stimulation method and topical anesthetic cream on pain and heart rate variability during arteriovenous fistula puncture in hemodialysis patients. Journal of Korean Biological Nursing Science. 2019;21(3):207-216. https://doi.org/10.7586/jkbns.2019.21.3.207

**A18.** Lee JO. Effects of a finger guard while opening the glass ampoule by nursing students. Journal of Korean Biological Nursing Science. 2019;21(4):318-325. https://doi.org/10.7586/jkbns.2019.21.4.318

**A19.** Kim M, Shin YK, Seol GH. Inhalation of clary sage oil before chemotherapy alleviates anxiety and stress without changing blood pressure: a randomized controlled trial. Journal of Korean Biological Nursing Science. 2021;23(4):267-275. https://doi.org/10.7586/jkbns.2021.23.4.267

**A20.** Her J, Hur MH. The effects of mask-applied aromatherapy on allergic rhinitis symptoms, fatigue, and quality of life related to allergic rhinitis in the COVID-19 era: a randomized controlled trial. Journal of Korean Biological Nursing Science. 2024;26(3):177-184. https://doi.org/10.7586/jkbns.24.021

**A21.** Lee SM, Hur MH. Thermoelectric tourniquet-assisted thermotherapy and cryotherapy for pain, regional blood flow, and satisfaction with intravenous injections among hospitalized patients in Korea: a randomized controlled trial. Journal of Korean Biological Nursing Science. 2024;26(4):323-336. https://doi.org/10.7586/jkbns.24.027

**A22.** Lee SH, Kim WJ, Choi EH, Hur MH. Comparative effects of music therapy and aromatherapy on stress, quality of life, and happiness among shift nurses in Korea: a randomized controlled trial. Journal of Korean Biological Nursing Science. 2024;26(4):337-349. https://doi.org/10.7586/jkbns.24.030
